# Hearing impairment in premature newborns—Analysis based on the national hearing screening database in Poland

**DOI:** 10.1371/journal.pone.0184359

**Published:** 2017-09-14

**Authors:** Katarzyna Wroblewska-Seniuk, Grazyna Greczka, Piotr Dabrowski, Joanna Szyfter-Harris, Jan Mazela

**Affiliations:** 1 Department of Newborns’ Infectious Diseases, Poznan University of Medical Sciences, Poznan, Poland; 2 Department of Otolaryngology and Oncological Laryngology, Poznan University of Medical Sciences, Poznan, Poland; Cincinnati Children's Hospital Medical Center, UNITED STATES

## Abstract

**Objectives:**

The incidence of sensorineural hearing loss is between 1 and 3 per 1000 in healthy neonates and 2–4 per 100 in high-risk infants. The national universal neonatal hearing screening carried out in Poland since 2002 enables selection of infants with suspicion and/or risk factors of hearing loss. In this study, we assessed the incidence and risk factors of hearing impairment in infants ≤33 weeks’ gestational age (wga).

**Methods:**

We analyzed the database of the Polish Universal Newborns Hearing Screening Program from 2010 to 2013. The study group involved 11438 infants born before 33 wga, the control group—1487730 infants. Screening was performed by means of transient evoked otoacoustic emissions. The risk factors of hearing loss were recorded. Infants who failed the screening test and/or had risk factors were referred for further audiological evaluation.

**Results:**

Hearing deficit was diagnosed in 11% of infants ≤25 wga, 5% at 26–27 wga, 3.46% at 28 wga and 2–3% at 29–32 wga. In the control group the incidence of hearing deficit was 0.2% (2.87% with risk factors). The most important risk factors were craniofacial malformations, very low birth weight, low Apgar score and mechanical ventilation. Hearing screening was positive in 22.42% newborns ≤28 wga and 10% at 29–32 wga and in the control group.

**Conclusions:**

Hearing impairment is a severe consequence of prematurity. Its prevalence is inversely related to the maturity of the baby. Premature infants have many concomitant risk factors which influence the occurrence of hearing deficit.

## Introduction

Childhood hearing impairment is the result of the overlapping factors of genetic predisposition and environmental impact. Worldwide reporting of hearing loss finds that the prevalence of moderate and severe bilateral hearing deficit (>50 dB) is 2–3 per 1000 live births in well baby nursery population and 2–4 in 100 infants in an intensive care population [1,2]. In Polish Universal Newborns Hearing Screening Program (PUNHSP) 3 per 1000 infants were diagnosed as having hearing problem, defined as any hearing impairment with threshold of 20 dB [3].

For effective treatment, congenital or perinatal hearing loss should be recognized within three months of birth so that early intervention can begin prior to 6 months of age. If not detected and hearing amplification provided, significant hearing impairment negatively impacts speech development and results in disorders in psychological and mental behavior [4].

The Joint Committee on Infant Hearing in 2000 listed 10 factors that identify infants at greatest risk for hearing impairment [4]. This list was updated in 2007 and such risk factors were added as treatment in the intensive care unit for more than 5 days and assisted ventilation [5]. Some studies report additional risk factors such as premature birth, low birth weight, respiratory distress syndrome and intracranial hemorrhage [2, 6–9]. In the questionnaire of the PUNHSP 12 risk factors are included (Table 1) [10].

**Table 1:** Risk factors for hearing impairment.

1. Family history of hearing loss
2. Craniofacial anomalies–anomalies of pinna, ear canal, ear tags, ear pits and temporal bones.
3. Complex congenital anomalies associated with congenital hearing loss
4. Congenital infections (TORCH infections, particularly cytomegalovirus)
5. Very low birth weight (<1500 g)
6. Premature birth (weeks < 33)
7. Hyperbilirubinemia requiring exchange transfusion
8. Ototoxic medications including but not limited to aminoglycosides used in multiple courses or in combination with loop diuretics such as furosemide
9. Bacterial meningitis
10. Low Apgar score—0–4 at 1 min or 0–6 at 5 min
11. Mechanical ventilation for at least 5 days
12. Intensive care > 7 days

The implementation of hearing screening programs for newborns continues to grow. Some institutions used to perform screening based on risk factors only [11–13]. However, it was proven that such screening protocol identifies only 50–75% of infants with hearing loss [11–13]. Therefore, it is now recommended to conduct universal hearing screening in all infants [5, 14].

Infant hearing screening is considered to be an effective procedure in early detection of hearing impairment in infants and therefore it should be one of the priorities in neonatal care [1, 4]. The goal of early hearing detection and intervention is to maximize linguistic competence and literacy development in children who have hearing impairment. To optimize the outcome of these children, the hearing of all infants should be screened at no later than 1 month of age [5].

The techniques most often employed and successfully used in the universal neonatal hearing screens are:

- Automated auditory brainstem response (ABR)
- Otoacoustic emissions (OAE).

Both OAE and automated ABR technologies provide noninvasive recordings of physiologic activity underlying normal auditory function and both are easily performed in neonates and infants [15].

Otoacoustic emissions recording usually takes less than 1 min and can be achieved without audiological knowledge [11]. The principle of the test is that the sound vibrations emitted from the cochlea flow to the ear canal where the acoustic energy is recorded. However, as OAEs are generated within the cochlea, OAE technology cannot be used to detect neural (eighth nerve and auditory brainstem pathway) dysfunction [15], that may result from exposure to ototoxic drugs or hyperbilirubinemia.

Auditory brainstem response is an auditory evoked potential that originates from the auditory nerve. It can detect injury on the level of cochlea, auditory nerve and auditory pathway in the brainstem. It is now recommended that infants admitted to the neonatal intensive care units (NICU) for more than 5 days have ABR included as part of their screening so that neural hearing loss will not be missed [5].

In Poland, the group of Polish neonatologists and otorhinolaryngologists, together with The Great Orchestra of Christmas Charity Foundation initiated the program of universal newborn hearing screening in 2002. This program is based on otoacoustic emissions testing and a questionnaire aimed at identifying risk factors defined by the Joint Committee on Infant Hearing published in 2000 [4], in all newborns prior to hospital discharge (1^st^ level of the hearing screening program). Infants in whom the result of OAE screen is positive, i.e. that receive the result ‘refer’ for at least one ear and/or those who have at least one risk factor of hearing impairment, are referred for further evaluation by means of ABR to the audiological centers responsible for hearing evaluation and appropriate intervention (2^nd^ level of the hearing screening program).

It is also now recommended that in all infants readmitted to the hospital in the first month of life, when there are conditions associated with potential hearing loss (eg. significant hyperbilirubinemia, culture-positive sepsis or bacterial meningitis), a repeat hearing screening is performed before discharge [5].

The group of infants that is particularly at risk of hearing deficit are premature newborns, born < 33 weeks of gestational age (wga), especially those treated in NICUs. Apart from congenital sensory or conductive hearing loss, these infants may develop sensory-neural hearing loss, known also as auditory neuropathy/auditory dyssynchrony, due to the treatment received in the NICU [5].

The aim of this study was to assess the incidence of hearing impairment in preterm infants (≤33 wga) and to analyze the risk factors of hearing loss in this population.

## Material and methods

We analyzed the records of the PUNHSP database from January 2010 till December 2013. In this time, a total of 1 499 168 infants were registered in the database. All infants were screened by means of transient evoked otoacoustic emissions (TEOAEs) and the risk factors of hearing loss were recorded in the questionnaire, filled in by medical staff based on the medical records and family interview (1^st^ level of the hearing screening program). Screening was performed with OtoRead screener (Interacoustics). The outcomes of screening were presented as either ‘refer’ (i.e. hearing problem detected in at least one ear) or ‘pass’ (i.e. hearing problem not detected). The list of risk factors used in the questionnaire was adopted from the JCIH statement of 2000 (Table 1) [4].

As described in detail previously [16], according to the protocol, healthy neonates were screened in the second or third day of life, whereas infants treated at the NICU–when their general condition was stable. All infants were tested bilaterally, without any sedation, while sleeping. If the ‘pass criteria’ of the first test were not achieved, the infant was rescreened in the same way on the day of discharge from the hospital. The result of the second test was treated as final. The screens were performed by the personnel trained in the use of the screening device.

Infants who failed the screening test and/or had risk factors of hearing impairment were referred to the outpatient audiology clinic for further evaluation within 3 months of life or immediately after hospital discharge (2^nd^ level of hearing screening program). Infants who did not have OAE screen performed on the 1^st^ level were also referred to the 2^nd^ level.

At the 2^nd^ level of PUNHSP infants were at first examined with otoscopy to determine the condition of the external auditory canal and tympanic membrane. Immittance audiometry measurement was performed: tympanometry and the registration of stapedius muscle reflex at frequencies of 500, 1000, 2000 and 4000 Hz. Infants were also screened once again with OAE using OtoRead screener (Interacoustics) and finally examined by means of auditory brainstem response method (Racia Alvar Centor C.). Absolute thresholds of hearing were defined using a click as stimulus and a specific stimulus for frequency 500 and 1000 Hz. Latencies of waves I, III and V were measured. Basing on the results, the diagnosed hearing deficit was classified as mild (21–40 dB HL), moderate (41–70 dB), severe (71–90 dB HL) and profound (>90 dB HL).

The hearing screening program covered more than 98% of infants born in Poland in this period of time (n = 1 525 094). These infants had at least one OAE screen performed and the questionnaire of risk factors filled in. The missing infants (n = 25 926) were not screened due to various logistic and medical situations.

The population was divided into two groups. The study group consisted of 11 438 infants born < 33 wga and was further divided into two subgroups:

- ≤28 wga–2884 neonates
- 29–32 wga–8554 neonates.

The control group consisted of 1 487 730 newborns born ≥33 wga.

**Fig 1:**
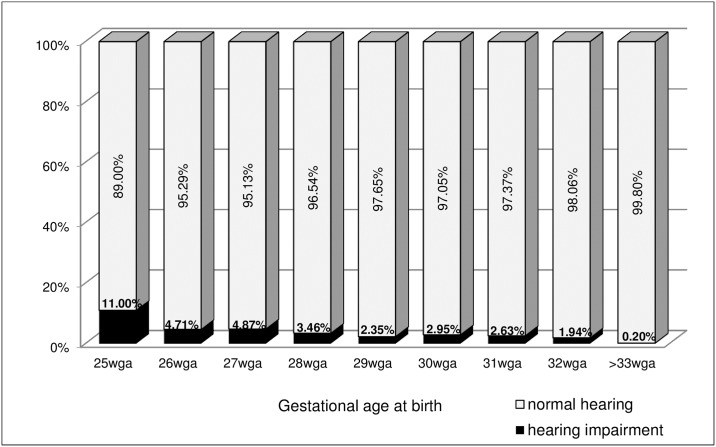
Flowchart of children registered in the central database of PUNHSP between 1 January 2013 and 31 December 2013. PUNSHP = Polish Universal Neonatal Hearing Screening Program; wga = week of gestational age.

All infants in the study group were treated as infants with risk factors of hearing impairment and independently of the screening result they were directed to the audiologists for further examination by means of ABR. Only about 55% of infants referred for further assessment showed up in the outpatient audiology clinics taking part in the neonatal hearing screening program. Among infants born before 33 wga it was 58.9%, thus a portion of our results is limited to the group of 6742 infants of the study group examined at the 2^nd^ level of screening program. In the control group (≥33 wga) only infants with risk factors of hearing impairment or with positive result of the screening OAE screen, were referred for further examination at the 2^nd^ level. Therefore, some of the analysis are limited only to those infants who were screened at 1^st^ level and examined at the 2^nd^ level of the program (n = 31348). Other infants of this group were treated as infants with normal hearing (negative screening test, no risk factors). Flowchart of children registered in the central database of PUNHSP between 1 January 2013 and 31 December 2013 with diagnostic tests performed is presented in Fig 1.

By terms hearing impairment and hearing deficit in PUNHSP we understand any permanent hearing problem either unilateral or bilateral of the level ≥20 dB, irrespectively of its etiology and time of diagnosis.

The STATISTICA 10 package was used for the analysis. Pearson Chi square, Odds Ratio (OR) and 95% confidence intervals (CI) were calculated. Multivariate logistic regression analysis was performed for the investigated risk factors and Cohen’s kappa coefficient for test agreement was calculated.

## Results

**Fig 2:**
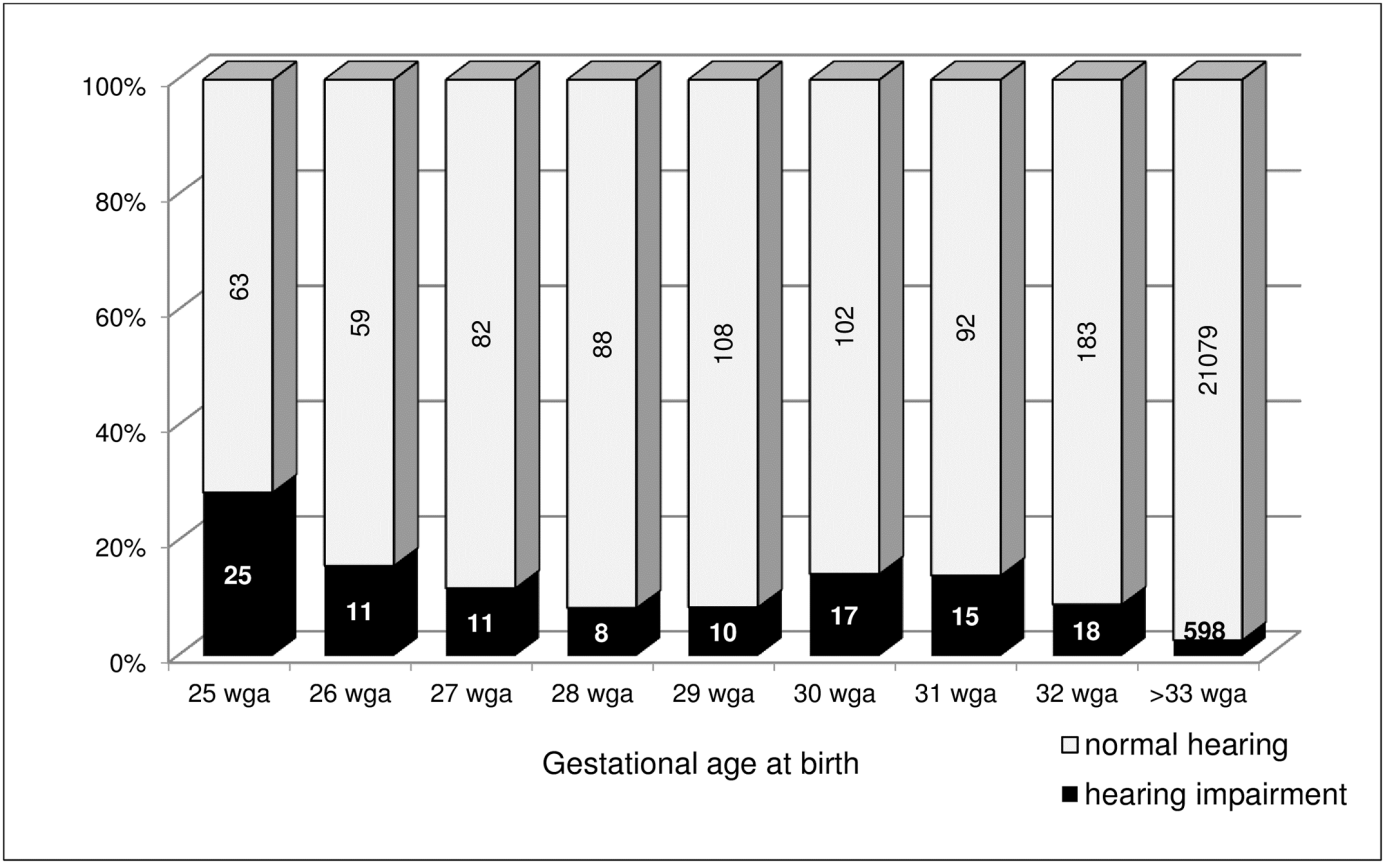
The prevalence of hearing impairment in relation to gestational age at birth. wga = week of gestational age at birth.

The final diagnosis of permanent hearing impairment was given predominantly in most premature infants, born ≤ 25 weeks of gestation– 11% (32 / 291). In infants born between 26 and 28 wga hearing deficit was diagnosed in 4.2% of patients (53 / 1257) and in those between 29 and 32 wga in 2.3% (122 / 5194)–Pearson Chi-square p<0.0001. The exact results for each week of gestation are shown on Fig 2. In the control group (≥33 wga) hearing deficit was diagnosed in 0.2% of examined infants (2950 / 1 487 730). However not all infants of the control group were rescreened at the second level of screening program by means of ABR, thus these results may only be treated as an estimation. We analyzed separately the infants of the control group in whom risk factors of hearing deficit were found and in this population, hearing deficit was diagnosed in 2.87% of patients.

We analyzed the incidence of different types and intensity of hearing deficits in relation to gestational age at birth. The results are presented in Table 2. The most serious problem–permanent profound sensorineural bilateral hearing deficit (> 90 dB) was diagnosed in 1.42% of infants born ≤ 28 wga (22/1548), in 0.3% of those born between 29 and 32 wga (16/5194) and in 0.02% of infants born ≥ 33 wga (p<0.01). Similar relation was observed also for other types and intensities of hearing deficit.

**Table 2:** Types and intensity of hearing deficits in relation to infants’ maturity.

		≤ 28 wga (n = 1548)		29–32 wga (n = 5194)		≥ 33 wga (n = 1 487730)		Total (n = 1494472)	
		Unilateral	Bilateral	Unilateral	Bilateral	Unilateral	Bilateral	Unilateral	Bilateral
**Sensorineural hearing loss**	≤ 40 dB	3	3	6	4	108	250	117	257
	41–70 dB	4	19	16	25	174	597	194	641
	71–90 dB	1	2	4	3	72	153	77	158
	≥ 90 dB	2	22	2	16	79	331	83	369
	**Total**	**10 (0.6%)**	**46 (3.0%)**	**28 (0.5%)**	**48 (0.9%)**	**433 (0.03%)**	**1331 (0.09%)**	**471 (0.03%)**	**1425 (0.1%)**
**Permanent conductive hearing loss**	≤ 40 dB	3	11	10	14	216	285	242	252
	41–70 dB	2	5	6	4	121	172	129	187
	71–90 dB	0	0	0	0	9	0	9	0
	≥ 90 dB	0	0	0	0	0	2	0	2
	**Total**	**5 (0.3%)**	**16 (1.0%)**	**16 (0.3%)**	**18 (0.3%)**	**346 (0.02%)**	**459 (0.03%)**	**380 (0.03%)**	**441 (0.03%)**
**Mixed type hearing loss**	≤ 40 dB	0	2	2	4	44	94	46	100
	41–70 dB	0	4	1	2	67	141	68	157
	71–90 dB	0	1	0	2	12	16	12	19
	≥ 90 dB	0	1	0	1	3	4	3	6
	**Total**	**0 (0)**	**8 (0.52%)**	**3 (0.06%)**	**9 (0.17%)**	**126 (0.01%)**	**255 (0.02%)**	**129 (0.01%)**	**282 (0.02%)**
	**Total**	**15 (1.0%)**	**70 (4.5%)**	**47 (0.9%)**	**75 (1.4%)**	**905 (0.06%)**	**2096 (0.14%)**	**991 (0.07%)**	**2177 (0.15%)**

The mean time of the final diagnosis in all groups of infants was 89^th^ day of life.

Among infants with permanent profound bilateral sensorineural hearing loss 349 received cochlear implants (94.6%). 897 children with other types of hearing deficit received hearing aids. Most children received also audiological, logopedical and psychological care.

Risk factors of hearing deficit were found in 64 580 (4.3%) infants. All infants born < 33 wga were treated as infants with risk factors due to their prematurity. At least one other risk factor was identified in 84.4% (n = 9651) of them. Most frequent risk factor was exposure to “ototoxic medications”, accounting for 63.0% in this population. The second and the third most frequent risk factors were “low birth weight <2500 g”– 53.3% and “treatment in the intensive care unit”– 43.9%. Table 3 presents the frequency of all risk factors with further division into two subgroups ≤28 wga and 29–32 wga versus control group ≥ 33 wga.

**Table 3:** The frequency of risk factors registered in the study and control groups.

Risk factors	≤28 wga		29–32 wga		≥ 33 wga	
	n	%	n	%	n	%
	2884	100	8554	100	1487730	100
**Family history of hearing loss**	6	0.21%	61	0.71%	9084	0.61%
**Craniofacial anomalies**	24	0.83%	64	0.75%	2139	0.14%
**Complex congenital anomalies associated with congenital hearing loss**	7	0.24%	22	0.26%	1199	0.08%
**Congenital infections (TORCH infections)**	41	1.42%	98	1.15%	12386	0.83%
**Very low birth weight (<1500 g)**	**2660**	**92.23%**	**3435**	**40.16%**	1176	0.08%
**Low Apgar score– 0–4 at 1 min**	914	31.69%	877	10.25	3726	0.25
**Low Apgar score—0–6 at 5 min**	428	14.84%	425	4.97%	930	0.06%
**Mechanical ventilation for at least 5 days**	**1657**	**57.45%**	**2000**	**23.38%**	2293	0.15%
**Intensive care > 7 days**	1919	66.54%	3101	36.25%	4646	0.31%
**Hyperbilirubinemia**	14	0.49%	20	0.23%	199	0.01%
**Ototoxic medications**	**2044**	**70.87%**	**5166**	**60.39%**	**25606**	**1.72%**
**Bacterial meningitis**	31	1.07%	61	0.71%	307	0.02%

The use of ototoxic medications was also the most frequent risk factor in the control group, however it was only recorded in 1.72% of infants born ≥ 33 wga. The second and third most frequent risk factors in the control group were congenital infections (0.83%) and family history of hearing loss (0.61%), however their frequency was comparable in the study group and was not significant.

Hearing evaluation in patients with specific risk factors revealed that in the group of infants born ≤ 28 wga in whom hearing impairment was diagnosed, the most important risk factors were craniofacial anomalies, low Apgar score at 1^st^ minute of life and mechanical ventilation. In infants born between 29 and 32 wga the most significant were craniofacial anomalies, complex congenital anomalies, extremely low birth weight and low Apgar scores. In the near term and term newborns (≥ 33 wga) the most important role played the family history of hearing loss, craniofacial anomalies and complex congenital anomalies, as well as very low birth weight and low Apgar scores. The exact data are shown in Table 4.

**Table 4:** The contribution of risk factors to hearing loss in infants of the study subgroups and the control group (multivariate logistic regression analysis—odds ratios (OR) and 95% confidence intervals).

Risk factors	≤28 wga			29–32 wga			≥ 33 wga		
	n	Hearing deficit		n	Hearing deficit		N	Hearing deficit^a^	
**TOTAL**	1548	85	5.5%	5194	122	2.3%	31348	931	2.97%
**Family history of hearing loss**	3	1	33.3%	40	3	7.5%	4560	223	4.9%
	OR 8.92 (CI 0.80; 99.48)			OR 2.14 (CI 0.57; 8.04)			OR 1.92* **(CI 1,7; 2.18)**		
**Craniofacial anomalies**	12	3	25%	16	3	18.8%	1019	254	24.9%
	OR 8.04* **(CI 2.23; 29.1)**			OR 4.81* **(CI 1.75; 13.24)**			OR 5.05* **(CI 4.48; 5.69)**		
**Complex congenital anomalies associated with congenital hearing loss**	2	0	0.0%	10	4	40.0%	585	144	24.6%
	OR ** 1			OR 36.24* **(CI 9.6; 136.87)**			OR 12.46* **(CI 10,14; 15.32)**		
**Congenital infections (TORCH infections)**	31	2	6.5%	77	1	1.3%	8462	72	0.9%
	OR 1.29 (CI 0.35; 4.72)			OR 0.62 (CI 0.09; 4.51)			OR 0.25 (CI 0.19; 0.31)		
**Very low birth weight** **(<1500 g)**	1434	83	5.8%	2100	63	3.0%	610	25	4.1%
	OR 2.95 (CI 0.71; 12.19)			OR 1.66* **(CI 1.13; 2.42)**			OR 1.51* **(CI 1.0; 2.29)**		
**Low Apgar score– 0–4 at 1 min**	464	37	8.0%	520	21	4.0%	2329	60	2.6%
	OR 2.11* **(CI 1.33; 3.35)**			OR 2.03* **(CI 1.24; 3.32)**			OR 0.88 (CI 0.67; 1.16)		
**Low Apgar score—0–6 at 5 min**	217	19	8.8%	262	13	4.9%	566	26	4.6%
	OR 1.53 (CI 0.85; 2.75)			OR 2.42* **(CI 1.31; 4.48)**			OR 1.57* **(CI 1.03; 2.39)**		
**Mechanical ventilation for at least 5 days**	937	68	7.3%	1181	36	3.3%	1255	49	3.9%
	OR 2.53* **(CI 1.44; 4.44)**			OR 1.28 (CI 0.84; 1.96)			OR 1.32 (CI 0.96; 1.81)		
**Intensive care > 7 days**	1116	69	6.2%	1924	50	2.6%	2668	94	3.5%
	OR 1.58 (CI 0.89; 2.81)			OR 1.05 (CI 0.71; 1.54)			OR 1.16 (CI 0.92; 1.47)		
**Hyperbilirubinemia**	11	0	0.0%	14	1	7.1%	135	3	2.2%
	OR** 1			OR 3.38 (CI 0.44; 26.09)			OR 0.81 (CI 0.26; 2.56)		
**Ototoxic medications**	1165	62	5.3%	3208	81	2.5%	16050	278	1.7%
	OR 0.78 (CI 0.47; 1.3)			OR 1.16 (CI 0.78; 1.73)			OR 0.41 (CI 0.36; 0,48)		
**Bacterial meningitis**	20	2	10%	43	2	4.7%	198	9	4.5%
	OR 2.24 (CI 0.5; 9.91)			OR 2.21 (CI 0.53; 9.25)			OR 1.55 (CI 0.76; 3.15)		

^a^. Not all infants of the control group were rescreened at the second level of screening program by means of ABR so this result may only be treated as estimation.

* p < 0.05

** These logit estimators use a correction of 0.5 in every cell of table used to compute statistics, that contain a zero.

**Fig 3:**
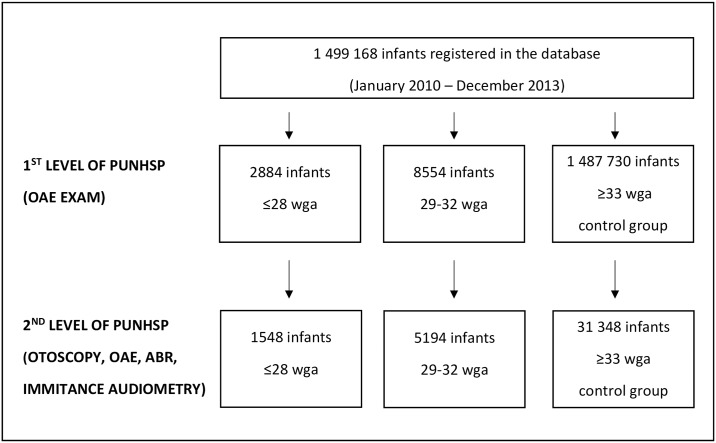
The percentage of patients with hearing deficit among infants with positive result of hearing screening.

Lastly, we analyzed the association between a positive result of the hearing screening and the final diagnosis of the hearing impairment. Fig 3 illustrates the number of infants in whom hearing screening suggested hearing deficits and the number of patients with the final diagnosis of hearing impairment according to the gestational age at birth.

Table 5 presents the data on the results of hearing screening and the final diagnosis of hearing deficit in the study subgroups. It shows that among most premature infants (≤28 wga), who have been examined on the second level of the PUNHSP, 22.4% had positive result of hearing screening test, that suggests hearing problems, and 15.8% of them were finally diagnosed with hearing deficit. In infants born between 29 and 32 wga as well as in the control group (≥ 33 wga) respectively 10.5% and 10% of infants failed hearing screening and out of that number, respectively 11% and 10% were diagnosed with hearing impairment requiring treatment. The agreement between the screening tests and the final diagnosis of hearing impairment was quite high in all three groups of patients (Table 5).

**Table 5:** The association between the positive result of the hearing screening and the final diagnosis of hearing deficit in the study subgroups.

	≤28 wga			29–32 wga			≥ 33 wga		
	N	Hearing deficit		N	Hearing deficit		N	Hearing deficit	
**Positive result of hearing screening**	347 (22.42%)	55	15.8%	545 (10.49%)	60	11%	21677 (10%)	2088	10%
Test agreement screening vs. final diagnosis*	**78.12%**			**89.12%**			**92.59%**		
	**p< 0.001**			**p<0.001**			**p<0.001**		

* Cohen’s kappa coefficient

There was also highly significant difference in the occurrence of hearing loss between those three groups (Pearson Chi-square, p = 0.00193).

## Discussion

Hearing impairment is an important and severe consequence of preterm birth and its prevalence is inversely related to the maturity of the infant. In our study, 4.2% of preemies born between 26 and 28 weeks of gestation and 2.3% of infants born between 29 and 32 wga were diagnosed with hearing impairment, similarly to the findings of other authors [1, 8, 17]. In infants born before 25 wga this problem was recognized even more frequently (11%) however due to the small number of patients in this subgroup the result may be overestimated. The incidence of profound bilateral sensorineural hearing deficit (1.42% ≤ 28 wga, 0.3% between 29 and 32 wga and 0.02% ≥ 33 wga) is also similar to the results of other researchers [1, 2, 8].

The pathophysiology of hearing loss in preterm infants is very complex and although prematurity alone may not have a severe impact on hearing, it is commonly associated with multiple other risk factors that can influence hearing in a synergistic fashion. Therefore, the risk of hearing loss in preemies is substantially higher than in the general newborn population.

There are many different known causes of neonatal hearing loss. It is generally accepted that genetic and environmental factors are each responsible for half of the cases [18, 19]. In our study, the family history of hearing deficit and congenital anomalies associated with hearing loss were not reported very frequently, however they seem to contribute significantly to the diagnosis of hearing loss. Craniofacial anomalies were significant risk factor in all three analyzed groups of patients, independently of their maturity. Other congenital defects were significant in infants born between 29 and 32 wga as well as in those born ≥33 wga. On the contrary, family history of hearing loss was important only in the group of most mature infants.

Other causes particularly important for hearing loss among premature infants include ototoxic drugs: aminoglycosides and loop diuretics, as well as noise exposure, hyperbilirubinemia and hypoxia [19]. Aminoglycosides in association with β-lactams are often treated as the first line antibiotics in newborns and are widely used in the NICUs. Unfortunately, they are known to damage both the cochlear and vestibular organs and produce irreversible hearing impairment by causing hair cell death. The damage to hair cells from aminoglycosides affects initially high-frequency hearing and progresses to involve lower frequencies [19, 20]. Ototoxicity of aminoglycoside depends on treatment duration, serum peak and trough concentrations, concomitant diseases and simultaneous administration of loop diuretics and vancomycin. Loop diuretics lead initially to reversible hearing loss by blocking ion transport within stria vascularis in cochlea. However, they increase also the rate of aminoglycoside-induced permanent hearing loss [19]. In our study exposure to ototoxic medications was among the most frequently reported risk factors in all subgroups. However, it did not seem to be significant.

Exposure to the constant background noise generated by contemporary life-support equipment in the NICU is another risk factor of hearing loss [19]. Recent studies suggest that free-radicals’ formation can be the underlaying mechanism of this pathology [20–21]. In our study 66.54% of infants ≤ 28 wga and 36.25% of infants born between 29 and 32 wga were treated in NICU for more than 7 days and respectively 57.45% and 23.38% of them were on mechanical ventilation for at least 5 days. Robertson et al. showed that mechanical ventilation and prolonged oxygen supplementation were associated with high prevalence of permanent hearing loss in extremely premature infants [22]. Hille et al. similarly presented that assisted ventilation ≥ 5 days is an independent risk factor for hearing loss [8]. Likewise, in our study mechanical ventilation lasting more than 5 days was a strong predictor of hearing impairment in most premature newborns.

Hyperbilirubinemia which can produce selective injury of the brainstem auditory nuclei and may damage the auditory nerve and ganglion cells [19, 23] does not seem to be an important risk factor for hearing loss in our study group. This might be due to the fact that nowadays we start treatment of hyperbilirubinemia by means of phototherapy very quickly without waiting for significant level of bilirubin. None of the patients of the study group required exchange transfusion. Other authors on the contrary found that in preterm infants the relationship between hyperbilirubinemia and hearing loss is important and is modified by other risk factors such as birth weight, mean duration of hyperbilirubinemia and acidotic incidents [19, 24]. De Vries et al found that among preterm infants with hyperbilirubinemia, those with very low birth weight (≤ 1500 g) have a higher risk of deafness than healthy infants with birth weight > 1500 g [24].

Hypoxia is strongly associated with hearing loss, as adequate oxygenation and perfusion are crucial for normal cochlear function [19, 25]. It has been revealed in some studies that severe birth asphyxia is an independent risk factor for hearing loss [8]. It is known that severe hypoxia may cause irreversible injury to the outer hair cells and stria vascularis in the cochlea, however there is no clear threshold level of hypoxia at which hearing might be destructed [25]. It may explain why low Apgar scores at birth and mechanical ventilation in NICU are associated with the risk of hearing deficit, which was confirmed also in our study.

According to our analysis, an important risk factor for hearing deficit is very low birth weight (VLBW < 1500 g). We could see this clearly in infants born between 29 and 32 wga and in those born ≥33 wga, where its significance was confirmed by the statistical analysis, however we must realize that in most premature newborns it was almost a constant feature (92.23%). Some studies also show this association while others do not [19, 26], which depends on the model of analysis, as VLBW is commonly related with other factors of hearing impairment.

It is known from many studies that some infants treated in NICU may begin to develop hearing impairment at the age of 2–4 years [27]. The pathophysiology of this delayed process is unclear; however, it may be caused by demyelinization or degeneration at points along the auditory pathway [27]. In our study, we did not assess infants for so long thus our reports might slightly underestimate the prevalence of hearing deficit in preterm infants.

In our study, we tried to analyze the prevalence of hearing deficit and the risk factors influencing hearing impairment in premature infants. Although involving large number of patients, the study has some limitations. First one is the referral rate of infants to the second level of hearing screening program which is about 60% and thus does not allow to perform the complete analysis. When discharged from the hospital parents were given written referral note to the 2^nd^ level of hearing screening and were explained about the importance of further audiological tests and rehabilitation. We think that some of these children were eventually diagnosed and treated in institutions that do not take part in the national program of hearing screening and that is why they are missing but we also suppose that some parents simply ignored the risk of hearing deficit and did not keep the scheduled appointments for their baby’s further assessment.

The study was based on the database of the national program and data was collected from 405 neonatal centers (1^st^ level of hearing screening). We know from our other study that in some cases there might be mistakes in entries to the database, which in consequence cause underestimation of the data on the percentage of follow-up visits at the 2nd level of PUNHSP [28]. However, this study also showed that parents of children who failed to meet the OAE pass criteria and/or had risk factors of hearing loss most frequently came for further diagnostics [28]. Other researchers have shown in their studies that main reasons for not attending follow-up visits are: insufficient number of pediatric audiology specialists, distance to the place of living, no referral, insufficient monitoring by pediatricians and inadequate knowledge of parents, who are not always able to notice clinical signs of hearing loss [29,30]. To improve the results of our program we should implement systems for reminding or notifying parents about the need to attend diagnostic level appointments, which are used in other countries.

The construction of the program makes it difficult also to compare the group of preterm and term infants. All premature newborns (<33 wga) are referred to the second level of screening where ABR and other exams are performed, while term infants are screened only once before discharge from the hospital and if they have no risk factors, they are treated as infants with normal hearing. Therefore, in our statistical analysis we could include only 31348 infants born ≥33 wga. These were infants who were examined at the second level of the hearing screening program and not the whole population of near term and term infants. We realize however that this limitation might change the picture of the whole population.

As stated before, the questionnaires of risk factors were filled in by medical staff before infants were discharged from the hospital after birth, based on the medical records of the patients. We admit however, that there might be some discrepancies between hospitals especially when reporting such risk factors as “the use of ototoxic drugs” or “hyperbilirubinemia”, which are not precisely defined in terms of duration or peak level.

Another limitation of our study might be the fact that we used only otoacoustic emissions as screening test, while it is suggested by the experts that all infants treated in NICU should be tested by means of auditory brainstem response [5]. However, we would like to underline that the PUNHSP is constructed in such way that all infants born <33 wga and all infants with other risk factors of hearing deficit have ABR test performed at the 2^nd^ level of screening. These children, independently of the results of OAE screen are referred to the 2^nd^ level center where they have all necessary diagnostic tests performed and the final diagnosis is given. Additionally, when we checked the agreement between the screening test and the final diagnosis of normal or impaired hearing, the results were very good–above 78% in infants ≤28wga and almost 90% in those born between 29 and 32 wga. Anyway, it is planned in the nearest future to introduce ABR in some NICU departments and perform the pilot study using these both techniques of screening.

The important result of the PUNHSP is that nearly all infants with permanent severe bilateral sensorineural hearing deficit obtained cochlear implants and most other infants received other hearing aids. All children were also included in audiological, logopedical and psychological care program.

The mean time of the final diagnosis and intervention in all groups of infants was 89^th^ day of life. This is appropriate time according to the standards of care and it enables an early intervention within 6 months of age [5, 31].

Following extreme prematurity, hearing impairment is a major adverse outcome that is commonly associated with other severe disabilities. What is also important this pathology may be postponed in time as delayed-onset and progressive hearing loss is not uncommon in this group of patients.

The extent to which prematurity alone is responsible for high prevalence of hearing impairment remains unclear. However, these patients are commonly exposed to other risk factors for hearing loss such as low Apgar score, intensive care treatment with mechanical ventilation, hypoxia, ototoxic drugs and hyperbilirubinemia. Therefore, long-term careful monitoring and the appropriate audiological management of hearing loss is essential among very premature infants.

## Conclusions

1. Hearing impairment is a severe consequence of prematurity and its prevalence is inversely related to the maturity of the baby.
2. Premature infants have many concomitant risk factors which influence the occurrence of hearing deficit. The most important of them seem to be low Apgar scores, mechanical ventilation, very low birth weight and craniofacial anomalies.
3. Some risk factors, e.g. “ototoxic drugs”, although frequently recorded, does not seem to be very significant.
